# Salt-Templated Nanoarchitectonics of CoSe_2_-NC Nanosheets as an Efficient Bifunctional Oxygen Electrocatalyst for Water Splitting

**DOI:** 10.3390/ijms23095239

**Published:** 2022-05-07

**Authors:** Hong Cao, Hailong Li, Linhao Liu, Kangning Xue, Xinkai Niu, Juan Hou, Long Chen

**Affiliations:** 1 Key Laboratory of Ecophysics, Department of Physics, College of Science, Shihezi University, Shihezi 832003, China; c_hong1002@sina.com (H.C.); x-15937874475@sina.com (K.X.); niuxinkai0424@sina.com (X.N.); 2 Key Laboratory for Green Process of Chemical Engineering of Xinjiang Bingtuan, School of Chemistry and Chemical Engineering, Shihezi University, Shihezi 832003, China; thebestleo@sina.cn (L.L.); chenlong2012@sinano.ac.cn (L.C.)

**Keywords:** metal–organic frameworks, nitrogen-doped carbon, CoSe_2_, oxygen reduction reaction, hydrogen evolution reaction

## Abstract

Recently, the extensive research of efficient bifunctional electrocatalysts (oxygen evolution reaction (OER) and hydrogen evolution reaction (HER)) on water splitting has drawn increasing attention. Herein, a salt-template strategy is prepared to synthesize nitrogen-doped carbon nanosheets encapsulated with dispersed CoSe_2_ nanoparticles (CoSe_2_-NC NSs), while the thickness of CoSe_2_-NC NSs is only about 3.6 nm. Profiting from the ultrathin morphology, large surface area, and promising electrical conductivity, the CoSe_2_-NC NSs exhibited excellent electrocatalytic of 10 mA·cm^−2^ current density at small overpotentials of 247 mV for OER and 75 mV for HER. Not only does the nitrogen-doped carbon matrix effectively avoid self-aggregation of CoSe_2_ nanoparticles, but it also prevents the corrosion of CoSe_2_ from electrolytes and shows favorable durability after long-term stability tests. Furthermore, an overall water-splitting system delivers a current density of 10 mA·cm^−2^ at a voltage of 1.54 V with resultants being both the cathode and anode catalyst in alkaline solutions. This work provides a new way to synthesize efficient and nonprecious bifunctional electrocatalysts for water splitting.

## 1. Introduction

Electrochemical water splitting has been an effective approach to generate sustainable and clean H_2_ energy [1,2,3,4]. To accelerate the production of hydrogen, numerous endeavors have been attempted to explore advanced and stable electrocatalysts that lower the overpotential of OER and HER [5,6,7]. Thus far, precious metal-based catalysts (such as Ir-based for OER and Pt-based for HER) are still the most effective oxygen electrocatalysts, but scarcity and high costs have largely hindered commercial applications. Hence, numerous research endeavors have been devoted to finding non-precious metal alternatives, including transition metal phosphates [8], nitrides [9], dichalcogenides [10,11], and borides [12,13,14], which showed exceptional activity and stability for OER and HER. Among them, cobalt selenide is a promising catalyst candidate. Its intrinsic metallic properties lead it to have higher electrical conductivity and more active edge sites [15,16,17]. For example, Lan et al. fabricated CoSe_2_ spheres via a facile hydrothermal process and the prepared samples exhibited an excellent electrochemical activity toward OER and HER [18].

In order to achieve higher electrochemical catalytic properties and stability for CoSe_2_, utilizing a nitrogen-doped carbon material as a supporter to enable CoSe_2_ particles is a feasible and widely used method. Metal organic frameworks (MOFs) have shown considerable advantages as precursors, and the well-organized framework consists of inorganic metal ions or clusters and N-containing organic ligands that can be converted in situ to nitrogen-doped carbon through pyrolysis [19,20,21,22]. Dong et al. found that pyrolysis and selenization of in situ grown zeolitic imidazolium framework-67 (ZIF-67) can homogeneously anchor CoSe_2_ nanoparticles (CoSe_2_/CF) to carbon fiber paper and the obtained CoSe_2_/CF shows excellent long-term stability and electrocatalytic properties [23]. However, metal sites in the MOF usually induce shrinkage agglomeration during high temperature calcination, which would disrupt the structure of the MOF and prevent exposure of the active site. Jiao et al. developed the SiO_2_ as templates to inhibit the Fe agglomeration during pyrolysis [24]. Notably, compared with the bulk counterparts, two-dimensional (2D) MOF nanosheets are being increasingly studied in electrocatalysis due to their highly exposed active sites [25], large surface area [26], and enhanced conductivity [27]. To date, synthetic methods of 2D MOF nanosheet preparation usually depends on physical exfoliation and chemical vapor deposition (CVD). Tang et al. prepared a series of ultrathin Ni/Co MOF nanosheets with unsaturated coordination metal active sites by a simple ultrasonic method, and demonstrated that ligand-unsaturated metal atoms are the main active centers of electrocatalytic OER. Nevertheless, how to avoid the aggregation and restacking of exfoliated nanosheets is still a challenge. Wurster et al. engineered heterobimetallic catalysts via CVD and the obtained nanosheets exhibited 300 mV overpotential and high turnover frequencies for OER [28]. Considering the low-yield of traditional methods, Huang et al. developed a salt-template confined method to prepare ultrathin ZIF-67 nanosheets [29]. The Co, N co-doped ultra-thin graphene nanosheets exhibited better electrocatalytic performance than commercial Pt/C catalysts. Thus, there is an urgent demand to develop a simple and cost-effective approach for designing 2D MOF derived efficient electrocatalysts with large surface area and fast mass transfer.

Here, CoSe_2_-NC nanosheet electrocatalysts were prepared using NaCl as a template. Inorganic salt has excellent chemical stability and a smooth surface, which is suitable as a template to build 2D structures. During pyrolysis, the outer layer of ZIF-67 served as the nitrogen source and carbon source for the in situ synthesis of nitrogen-doped carbon. The resultant compounds showed a higher electrochemically active surface area (ECSA) and stability. It also displays excellent OER and HER activity. In addition, the water-splitting cell, based on CoSe_2_-NC bifunctional catalysts, shows good electrochemical performance, demonstrating that the catalysts with 2D MOF-derived nanosheet structures have great potential for practical applications.

## 2. Results and Discussion

### 2.1. Characterization of CoSe_2_-NC NSs

**Figure 1:**
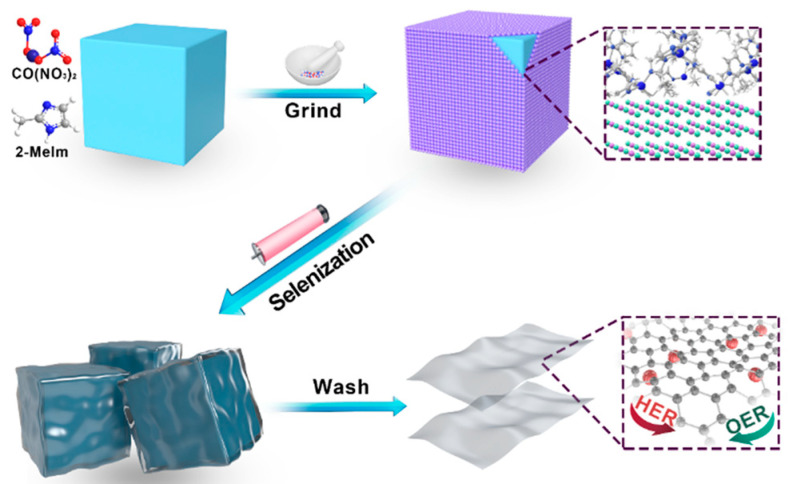
Schematic illustration of the overall synthetic procedure of CoSe_2_-NC NSs.

Commercial NaCl powder was selected as template for the one-step synthesis of 2D CoSe_2_ nanosheets. A schematic diagram of the material fabrication process is shown in Figure 1. First, NaCl powder was mixed with cobalt (II) nitrate and 2-methylimidazole precursors. After vigorous grinding, the organic ligands of imidazole coordinate to Co^2+^ at room temperature (Figure S1). It should be noted that, in this approach, the excess of NaCl is used to avoid the aggregation of Co during selenization. The EDS images of the NaCl@MOF (Figure S2) show the uniform growth of ZIF-67 on the NaCl surface. SEM image of the 2D MOF which removed the salt template (Figure S3) evidenced that the salt template successfully synthesized ZIF-67 nanosheets. The diffraction peaks of NaCl@MOF and NaCl@CoSe_2_ (Figure S4) both match the NaCl crystallinity, and no other peaks were displayed, indicating that the formed ZIF-67 layer was relatively thin. Afterwards, the ZIF-67 shell and Se powder were calcined at 750 °C in a N_2_-protected tube furnace and converted to CoSe_2_-NC. 

**Figure 2:**
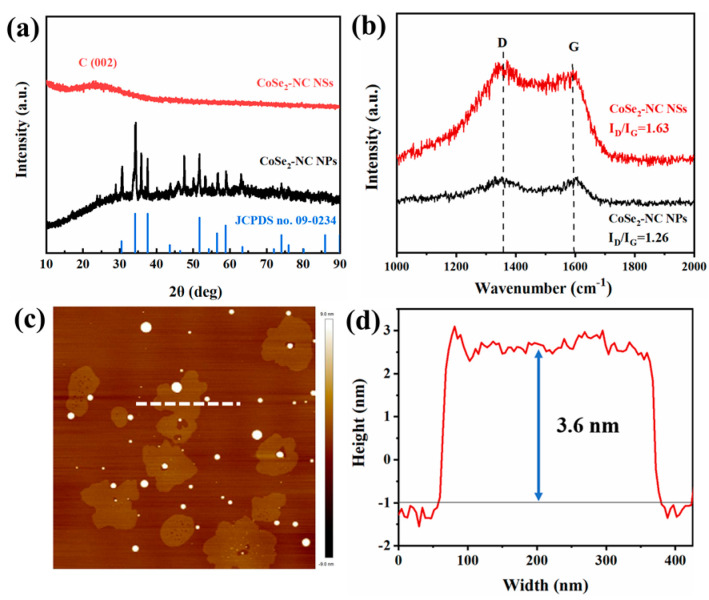
(**a**) XRD pattern of CoSe_2_-NC NSs and CoSe_2_-NC NPs, (**b**) Raman spectra of CoSe_2_-NC NSs and CoSe_2_-NC NPs, (**c**,**d**) AFM image of CoSe_2_-NC NSs.

To further expand the applicability of the synthetic method, amorphous nanosheets with a thickness of about 3.6 nm were effectively corroborated by XRD, Raman, and AFM. As shown in Figure 2, the nanosheets showed no obvious diffraction peaks, indicating that CoSe_2_-NC NSs are amorphous materials [30]. The graphite (002) peak in CoSe_2_-NC NSs implied graphitization. Compared with the 3D counterpart, the diffraction peaks of CoSe_2_-NC NPs were a perfect match with the simulated patterns (JCPDS, No. 09-0234), suggesting high crystallinity. As is known, the activity and number of exposed active sites directly affect the activity of electrocatalysts. Compared to its crystalline counterpart, the non-crystalline structure possesses more unsaturated coordination sites and effective active sites. Raman spectroscopy (Figure 2b) showed two peaks. The peak at 1355 cm^−1^ was due to the disordered sp3 carbon (D-band) and the peak at 1580 cm^−1^ indicated the existence of graphite sp2 carbon (G-band) [31]. Typically, the G-band corresponds to the lattice characteristics of graphite, while the D-band corresponds to the vibrational modes of carbon atoms at the edges of graphene [32,33]. The degree of carbon disorder is usually estimated by the value of I_D_/I_G_ [34,35]. The calculated ratios of I_D_/I_G_ were 1.63 for CoSe_2_-NC NSs and 1.26 for CoSe_2_-NC NPs. The results showed that the salt template prepared nanosheets with a higher I_D_/I_G_ than the 3D structure, which indicated that the nanosheets had abundant defects and were considered catalytically active sites [36,37]. The AFM images (Figure 2c) were also used to evaluate the thickness of CoSe_2_-NC NSs. As revealed in the AFM images, Figure 2d suggests that CoSe_2_-NC NSs exhibited ultrathin nanosheets with a thickness of 3.6 nm. Ultrathin nanosheets can expose abundant catalytic active sites during the OER and HER multiphase reaction interface.

**Figure 3:**
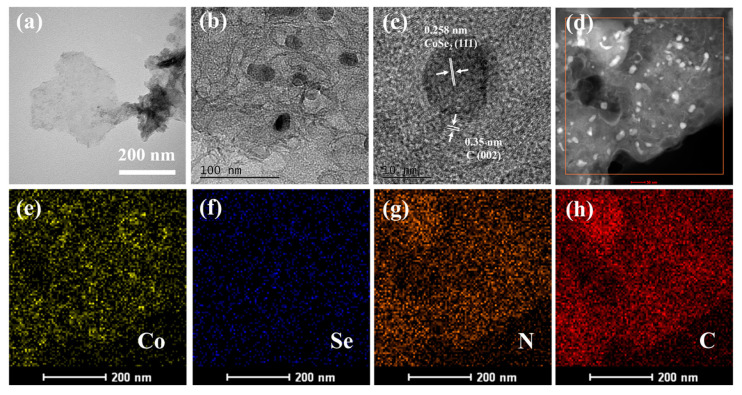
(**a**,**b**) TEM images of the CoSe_2_-NC NSs, (**c**) HRTEM image of the CoSe_2_-NC NSs, (**d**–**h**) EDS elemental mapping images of CoSe_2_-NC NSs.

To obtain more details of the structure, the morphologies of the CoSe_2_-NC NSs were observed using TEM. Figure 3a demonstrates that CoSe_2_-NC was in a sheet-like morphology. According to Figure S6, the dodecahedral shapes of the CoSe_2_-NC NPs could be well preserved, with dimensions of around 500 nm. Figure 3b shows that the CoSe_2_ particles were densely interconnected with the graphene layers. HRTEM showed that the interplanar distance of the lattice fringes was 0.258 nm, corresponding to the (111) plane of CoSe_2_, and 0.35 nm, corresponding to the (002) plane of graphite [38]. Moreover, the elemental mapping images (Figure 3d–h) verified that abundant C and N distributed throughout the entire sample. The results indicated the CoSe_2_ particles were encapsulated in nitrogen-doped carbon layers, which can induce greater stability during the corrosion of electrolytes.

### 2.2. Electronic States of CoSe_2_-NC NSs

**Figure 4:**
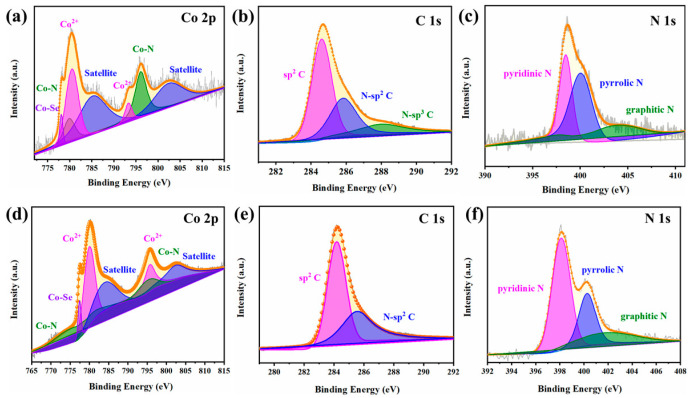
(**a**–**c**) High-resolution XPS spectra of Co 2p, C 1s and N 1s, for the CoSe_2_-NC NSs, (**d**–**f**) High-resolution XPS spectra of Co 2p, C 1s, and N 1s for the CoSe_2_-NC NPs.

The elemental compositions of CoSe_2_-NC NSs were determined by XPS analysis. The XPS spectrum of Co 2p (Figure 4a) can be divided into Co 2p3/2 and Co 2p1/2, which were located at 780.7 and 796.6 eV, and the corresponding satellite peaks were at 786.5 eV and 803.2 eV, respectively. These results indicated the presence of Co^2+^ [39]. The measured binding energy of 778.1 eV, relative to the reported binding energy of metallic Co, indicated that the Co on the catalyst surface was oxidized by Se elements [40]. The measured binding energy of 778.1 eV, relative to the reported binding energy of metallic Co, indicated that Co on the catalyst surface was oxidized by Se elements [41]. Pyridinic N and pyrrolic N can both coordinate with Co, so the peaks at 781.8 and 797.8 eV could be assigned to Co-N structures. In the Se spectral region (Figure S10), the two main characteristic peaks of CoSe_2_-NC NSs were located at 54.9 and 55.8 eV, which correspond to the Se 3d5/2 and 3d3/2 orbitals of Se^2−^, respectively. In addition, the peak located at 60.1 eV indicated the presence of Se-O bonds on the surface. These relative peaks were from Co^2+^ coordinated to Se ions. The peaks of the C 1s spectrum (Figure 4b) at 284.3 eV, which could be assigned to sp2 hybridized carbons, and the peaks at 285.4 and 286.9 eV, due to the N-sp2 C and N-sp3, demonstrated the successful doping of N into carbon [42]. Notably, the C 1s spectrum of CoSe_2_-NC NPs (Figure 4e) could be only deconvoluted into sp2 C and N-sp2 C. The presence of the sp3 carbon atoms could disrupt the long-range order of the carbon network and were considered to be defective sites in the sp2 carbon matrix. The analysis results in Figure 4c show that the characteristic peaks were located at 398.4, 399.6, and 400.7 eV, corresponding to pyridine N, pyrrole N, and graphite N, further confirming the formation of nitrogen-doped graphitic carbon [43]. Figure 4f shows that the pyrrole N peak of cobalt selenide is prominent, indicating that the three-dimensional structure of CoSe_2_-NC NPs has more pyrrole N in the annealing process. However, the pyrrole N species are nitrogen atoms in a five-membered C-N heterocyclic structure, which are unstable due to their special structure. These results indicated that the CoSe_2_ particles were successfully encapsulated into N-doped carbon (NC) matrix. For increasing active sites, doping the carbon matrix with nitrogen heteroatoms is useful. In addition, the nitrogen formed a strong bond with the internal atoms, which resulted in a high stability of the composite [44].

### 2.3. Electrochemical Performance of CoSe_2_-NC NSs

**Figure 5:**
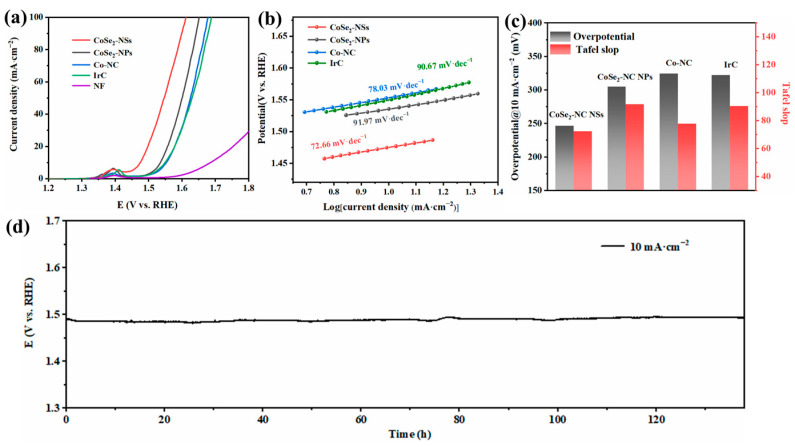
(**a**) LSV curves of CoSe_2_-NC NSs, CoSe_2_-NC NPs, Co-NC, IrC and NF, (**b**) Tafel slopes of CoSe_2_-NC NSs, CoSe_2_-NC NPs, Co-NC and IrC, (**c**) Tafel slopes and overpotential for OER of CoSe_2_-NC NSs, CoSe_2_-NC NPs, Co-NC and IrC, (**d**) stability of CoSe_2_-NC NSs in chronopotentiometry curve.

The performance of the OER catalysts were tested in 1 M KOH electrolyte. Typically, the potential of the OER at a current density of 10 mA·cm^−2^ is defined as E_j=10_. The overpotential is subtracted from the E_j=10_ value of the catalyst by 1.23 V. Figure 5a shows the LSV curves for the OER electrocatalytic performance of CoSe_2_-NC NSs, CoSe_2_-NC NPs, Co-NC, and IrC using a three-electrode configuration. It is a remarkable that the CoSe_2_-NC NSs catalyst exhibited an advanced electrocatalytic activity with an overpotential of 246.7 mV, which is evidently better than that of other catalysts, and is even better than IrC (overpotential = 322.8 mV. The Tafel slope of CoSe_2_-NC NSs (72.66 mV·dec^−1^), CoSe_2_-NC NPs (91.97 mV·dec^−1^), Co-NC (78.03 mV·dec^−1^), and IrC (90.67 mV·dec^−1^) are displayed in Figure 5b, and suggests that the morphology of the nanosheet directly increases OER activity. Figure 5c shows the overpotential and Tafel slope data of CoSe_2_-NC NSs, CoSe_2_-NC NPs, Co-NC, and IrC, indicating that CoSe_2_-NC NSs have a far greater OER activity than the other samples. The synergistic effect of CoSe_2_ and nitrogen-doped carbon contributes to the outstanding OER activity of CoSe_2_-NC NSs. Meanwhile, the stability of the catalyst in electrolyte is a key parameter. As shown in Figure 5d, CoSe_2_-NC NSs maintained a stable voltage at current density of 10 mA·cm^−2^ after 132 h of chronopotentiometry testing. The outstanding stability mainly originates from the carbon layers protecting the CoSe_2_ nanoparticles.

**Figure 6:**
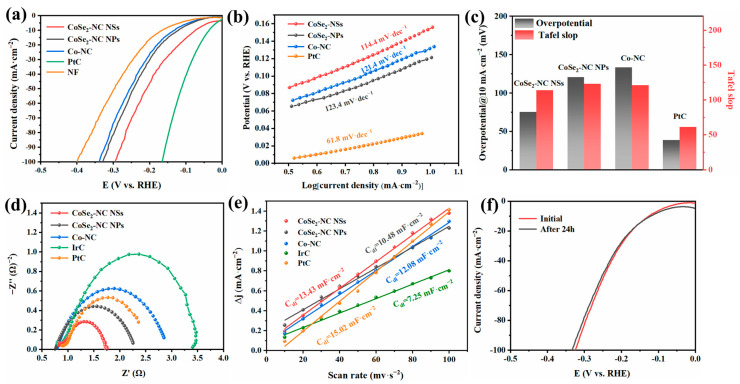
(**a**) LSV curves of CoSe_2_-NC NSs, CoSe_2_-NC NPs, Co-NC, PtC and NF. (**b**) Tafel slopes of CoSe_2_-NC NSs, CoSe_2_-NC NPs, Co-NC and PtC. (**c**) Tafel slopes and overpotential for HER of CoSe_2_-NC NSs, CoSe_2_-NC NPs, Co-NC and PtC. (**d**) C_dl_ curves of CoSe_2_-NC NSs, CoSe_2_-NC NPs, Co-NC, PtC and IrC. (**e**) EIS test of CoSe_2_-NC NSs, CoSe_2_-NC NPs, Co-NC, PtC and IrC. (**f**) LSV curves initial and after 24 h of continuous CV.

To assess the HER electrocatalytic activity, CoSe_2_-NC NSs, CoSe_2_-NC NPs, Co-NC, and PtC were explored in 1 M KOH electrolyte using a three-electrode cell. The CoSe_2_-NC NSs catalyst exhibited favorable electrocatalytic properties (Figure 6a). The overpotential of CoSe_2_-NC NSs was only 75.6 mV, which is much less than CoSe_2_-NC NPs (121.1 mV) and Co-NC (133.6 mV). The Tafel slope of 114.4 mV·dec^−1^ was measured for CoSe_2_-NC NSs, and was lower than CoSe_2_-NC NPs and Co-NC. As shown in Figure 6d, the ECSA of CoSe_2_-NC NSs (13.43 mF·cm^−2^) was much higher than CoSe_2_-NC NPs (10.48 mF·cm^−2^), Co-NC (12.08 mF·cm^−2^), and IrC (7.25 mF·cm^−2^). The results indicate that there were more active sites in CoSe_2_-NC NSs and that these abundant active sites were derived from the low-dimensional nanosheets, which have a large specific surface area (Figure S10). To further explore the HER kinetics, EIS measurements (Figure 6e) were taken in 1.0 M KOH. The smaller semicircle diameter of CoSe_2_-NC NSs (1.21 Ω) demonstrates a smaller charge-transfer resistance. This reveals that the electrochemical impedance of the CoSe_2_-NC NSs is much lower, which can effectively accelerate the charge transfer between the electrocatalyst and electrolyte interface.

**Figure 7:**
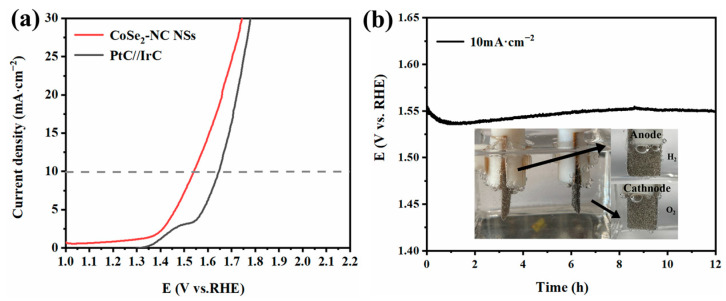
(**a**) LSV curves of the CoSe_2_-NC NSs and PtC||IrC with a two-electrode system in alkaline electrolyte. (**b**) Stability of CoSe_2_-NC NSs in 1 M KOH at a current density of 10 mA·cm^−2^ over 12 h.

In view of the high performance of the prepared CoSe_2_-NC NS electrodes for both OER and HER, a two-electrode cell was set up in 1.0 M KOH using CoSe_2_-NC NSs as both the cathode and anode. Figure 7a shows the LSV curves of the CoSe_2_-NC NSs, which exhibited excellent overall water splitting activity. For the LSV measurement, the cell voltage of the CoSe_2_-NC NSs-based water splitting cell at 10 mA·cm^−2^ was only 1.54 V, even below that of the PtC||IrC (1.65 V). After 12 h of constant current testing, the catalyst voltage showed no obvious change, which indicated that the catalyst has good electrochemical activity and stability. The salt template promotes the formation of ultrathin nanosheets. The strong bonding of CoSe_2_ and the carbon layer ensures the immobilization of the active component, which is beneficial for improving the durability of the electrochemical process for overall water splitting.

## 3. Materials and Methods

### 3.1. Materials and Reagents

Co(NO_3_)_2_·6H_2_O (99%), 2-methylimidazole (2-MeIm, 99%), and selenium powder (99.999%) were supplied by Sigma Aldrich (Missouri, USA). Methanol (99%), ethanol (99%), NaCl and KOH were bought from Chemical Reagent (Guangzhou, China). Nafion solution (5%) was purchased from Hesen (Shanghai, China). The ultrapure water (18 MΩ) used in the experiments was prepared using Hhitech equipment (Shanghai, China). Commercial catalysts (Pt/C, 20 wt%, Ir/C, 5 wt%) for comparison were bought from Macklin (Shanghai, China). All chemicals in the experiment were used directly without further purification.

### 3.2. Synthesis of Co-NC

A total of 1.97 g 2-methylimidazole was dissolved in a mixed solvent with 20 mL of methanol and 20 mL of ethanol. Meanwhile, 0.87 g Co(NO_3_)_2_·6H_2_O was dissolved in another mixed solvent with 20 mL of methanol and 20 mL of ethanol. Then, the above two solutions were mixed under continuous stirring for 1 min and the final solution was kept at room temperature for 24 h. The resultant purple ZIF-67 precipitate was collected using centrifugation and washed several times with ethanol and ultrapure water and then dried in an oven at 60 °C for 12 h.

### 3.3. Synthesis of CoSe_2_-NC NPs

The ZIF-67 particles and 0.1 g Se powder were dispersed in ceramic boats and the temperature in the furnace was raised to 750 °C at a rate of 2 °C·min^−1^. After that, the furnace was naturally cooled to room temperature. During the pyrolysis, the furnace was under a N_2_ atmosphere. To remove the free metal ions, the prepared black powder product was stirred in 0.5 M hydrochloric acid for 12 h. The samples were collected by centrifugation and washed repeatedly with deionized water and then dried at 80 °C.

### 3.4. Synthesis of CoSe_2_-NC NSs

First, 0.363 g Co(NO_3_)_2_·6H_2_O, 0.411 g 2-methylimidazole and 4 g NaCl salt were mixed and ground in a mortar. After that, ZIF-67 coated on NaCl nanocrystals surface (denoted as NaCl@ZIF-67) was obtained. Then, the as-obtained product and 0.1 g Se powder were selenized under a N_2_ atmosphere at 750 °C for 2 h, 2 °C·min^−1^. After being cooled, the powders were washed with 0.5 M HCl solution and deionized water to remove the NaCl templates and impurities.

### 3.5. Material Characterization

X-ray diffraction (XRD) was carried out using an X’ Pert PRO with a Cu Ka radiation diffractometer (k = 1.5418 Å). Raman spectra were detected using a Horiba JobinYvon, LabRAM HR800. Atomic force microscopy (AFM) images were determined using a Bruker Dimension ICON. The morphology and structures of the catalysts were measured by scanning electron microscopy (SEM, ZEISS Sigma 300) and an energy dispersive spectrometer (EDS). Transmission electron microscopy (TEM) was performed on a Tecnai G2 F30. The Brunauer–Emmet–Teller (BET) surface areas were measured at 77 K using a NOVA 2000 (Quantachrome, Boynton Beach, FL, USA). X-ray photoelectron spectroscopy (XPS) analyses were performed on a Thermo Scientific K-Alpha with Al Ka radiation.

### 3.6. Electrochemical Performance

Prior to catalyst loading, nickel foam was acid washed to remove oxide impurities from the surface. The 1 cm × 1 cm nickel foam was immersed in 1 M HCl solution and sonicated for 15 min, and then sonicated in deionized water and anhydrous ethanol solution for 15 min. A homogeneous suspension of the catalyst was formed by dispersing 2 mg of catalyst in a mixture of 250 µL of deionized water, 700 µL of anhydrous ethanol, and 50 µL of Nafion with sonication for 1 h. Then, the suspension (100 μL) was dripped onto the pre-polished nickel foam and dried in a vacuum at 60 ℃. The mass loading of the active materials in this paper was, on average, 0.2 mg·cm^−2^.

Electrocatalytic OER and HER measurements were tested at room temperature in a standard three-electrode setup, which was carried out on a CHI 760E electrochemical workstation (Chenhua, Shanghai, China). Nickel foam with electrocatalyst, graphite rod, and Ag/AgCl electrode filled with saturated KCl were selected as the working electrode, counter electrode, and reference electrode, respectively. The electrolyte used was KOH solution at 1.0 M (pH 13.6). To ensure the O_2_/H_2_O equilibrium at 1.23 V vs. RHE, all electrochemical experiments were performed in the O_2_ saturated condition. Linear sweep voltammetry (LSV) and cyclic voltammetry (CV) curves were measured at a scan rate of 5 mV·s^−1^. Tafel slopes were calculated according to Tafel equation: η = a + b log(j). CV was measured in the potential window of a non-Faraday process at different scan rates from 10 to 100 mV·s^−1^. The slope C_dl_ was obtained by fitting the current density versus scan rate as a linear relationship To further investigate electrocatalytic kinetics, electrochemical impedance spectroscopy (EIS) measurements were carried out in the frequency range 10 kHz to 10 mHz. The stability of the catalyst was determined by chronopotentiometry measurements at j = 10 mA·cm^−2^.

## 4. Conclusions

In summary, CoSe_2_ nanoparticles embedded into nitrogen-doped carbon nanosheets were successfully synthesized using a salt-template strategy. The ultrathin nanosheets formed by the salt could effectively avoid self-aggregation of the CoSe_2_ particles, while the 2D structure could promote more efficient electron transfer between reactants and catalysts. In comparison with CoSe_2_-NC NPs and bare Co-NC NPs, the CoSe_2_-NC NSs exhibited remarkable OER and HER properties. In addition, the robust structure can maintain excellent stability during the reaction. As a result, this work provides a new strategy for the design of CoSe_2_-based bifunctional electrocatalysts with excellent catalytic performance and long-term stability.

## Data Availability

Not applicable.

## Supplementary Materials

The following supporting information can be downloaded at: https://www.mdpi.com/article/10.3390/ijms23095239/s1.
