# Supplementary material for: Salt-Templated Nanoarchitectonics of CoSe2-NC Nanosheets as an Efficient Bifunctional Oxygen Electrocatalyst for Water Splitting
Source: Int J Mol Sci. 2022 May 7;23(9):5239. doi: 10.3390/ijms23095239 (PMC9099664; doi:10.3390/ijms23095239)
Supplement: Supplementary file 1 [file ijms-23-05239-s001.zip › ijms-1695556-supplementary.pdf]

Supplementary Information

Salt-templated nanoarchitectonics of $CoSe_2$-NC nanosheets as an efficient bifunctional oxygen electrocatalyst for water split-ting

Hong Cao [a], Hailong Li [a*], Linhao Liu [a,b], Kangning Xue [a], Xinkai Niu [a], Juan Hou [a*], Long Chen [b]

[a] College of Science/Key Laboratory of Ecophysics and Department of Physics, Shihezi University, Shihezi 832003, Xinjiang, China.

[b] School of Chemistry and Chemical Engineering/Key Laboratory for Green Process of Chemical Engineering of Xinjiang Bingtuan, Shihezi University, Shihezi 832003, Xinjiang, China.

*Corresponding Author.

Email address: well09131015@126.com

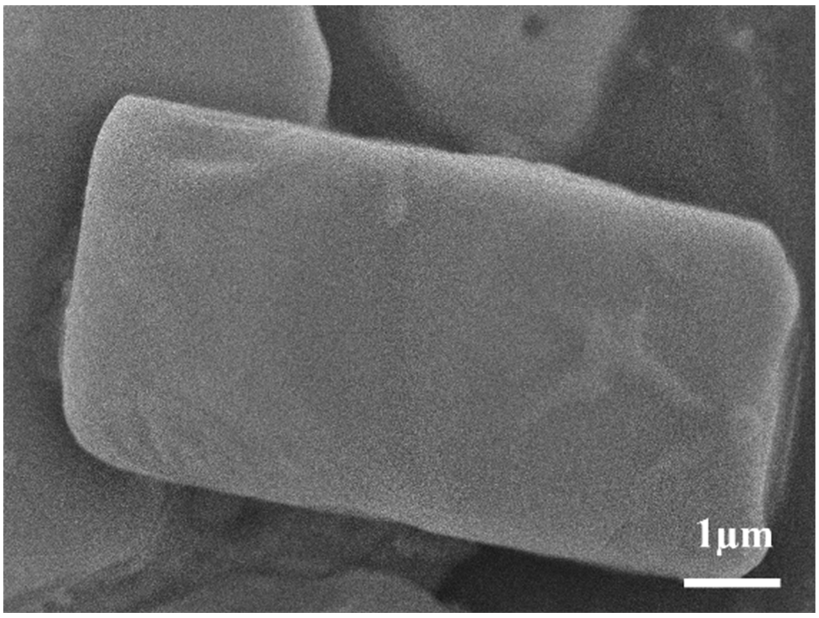

Figure S1: SEM image of the NaCl@MOF.

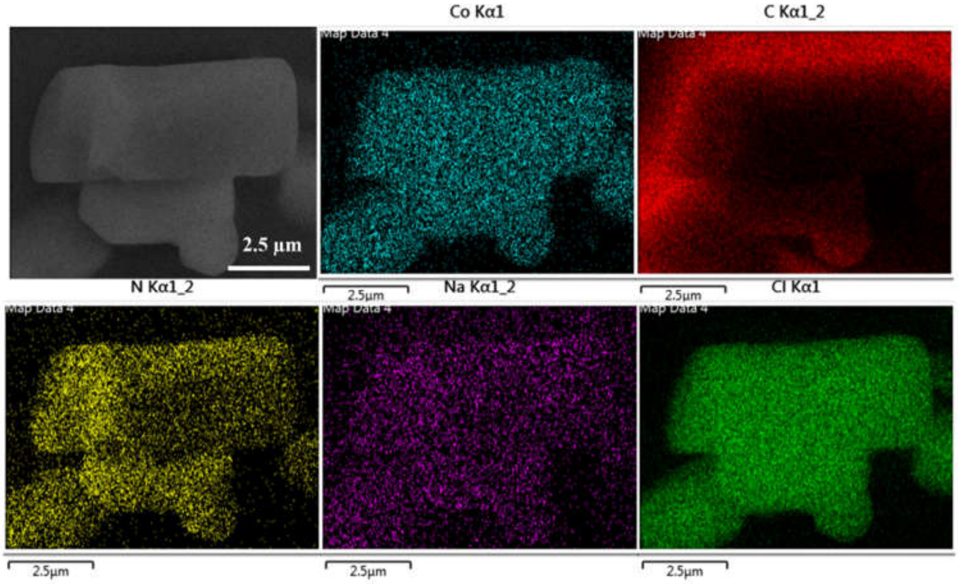

Figure S2: EDS elemental mapping images of NaCl@MOF.

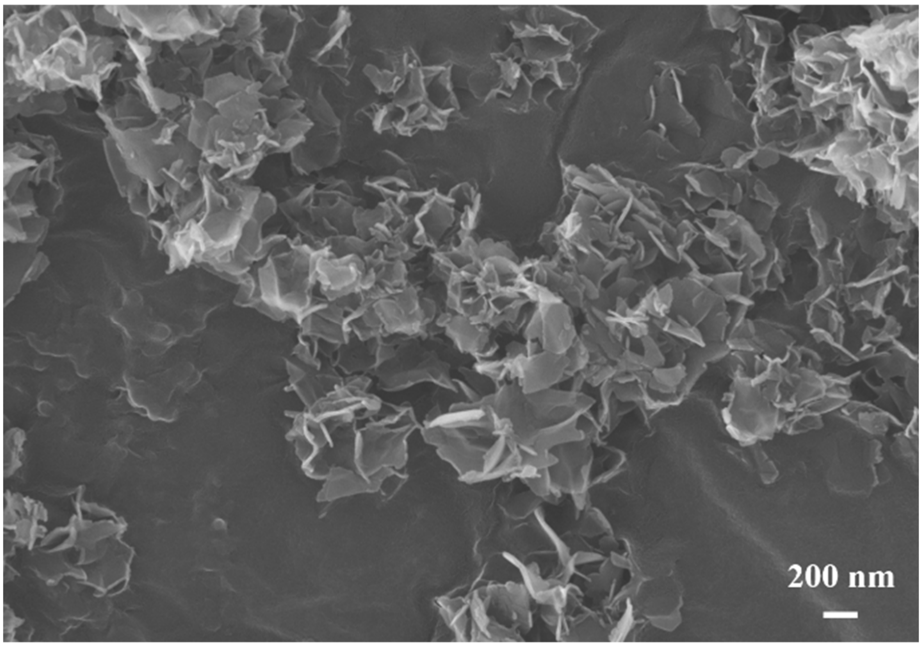

Figure S3: SEM image of the 2D MOF with removed salt-template.

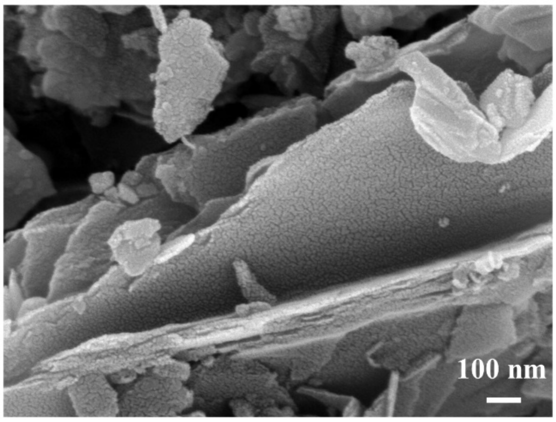

Figure S4: SEM image of $CoSe_2$-NC NSs.

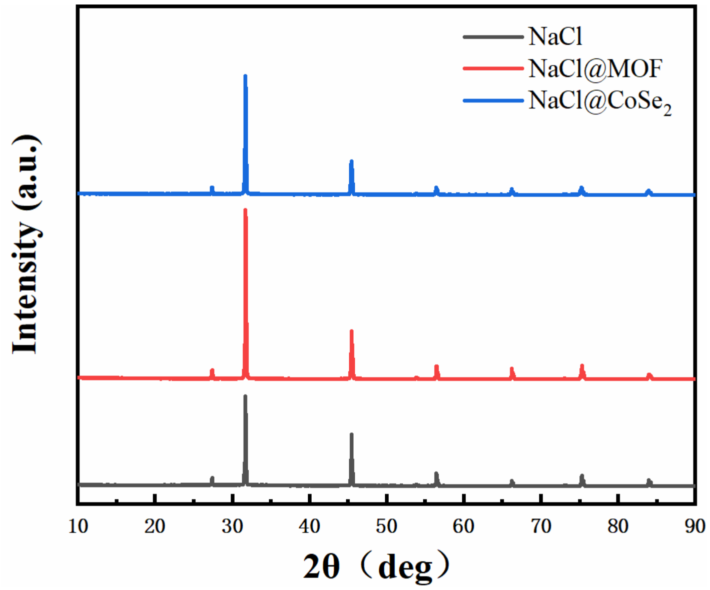

Figure S5: XRD pattern of NaCl crystal, NaCl@MOF, and NaCl@$CoSe_2$.

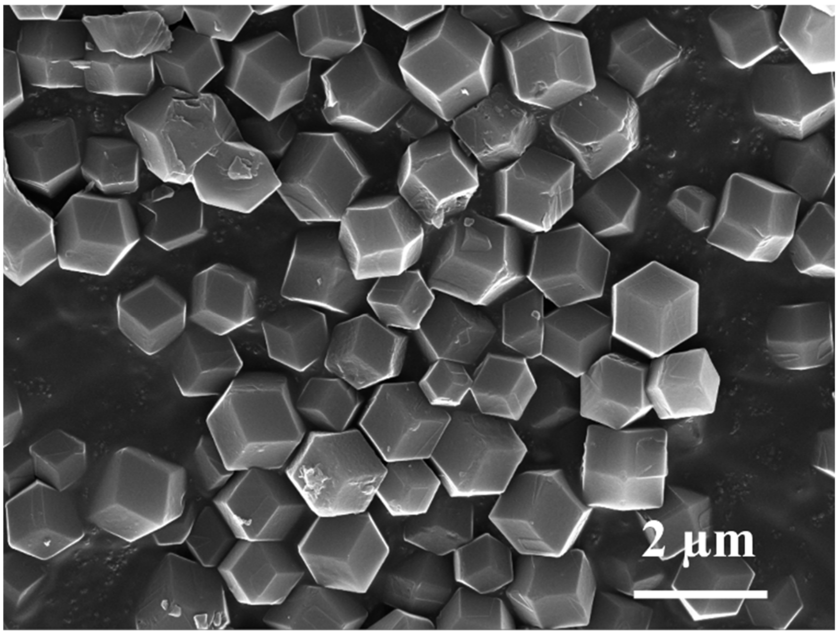

Figure S6:SEM image of the ZIF-67.

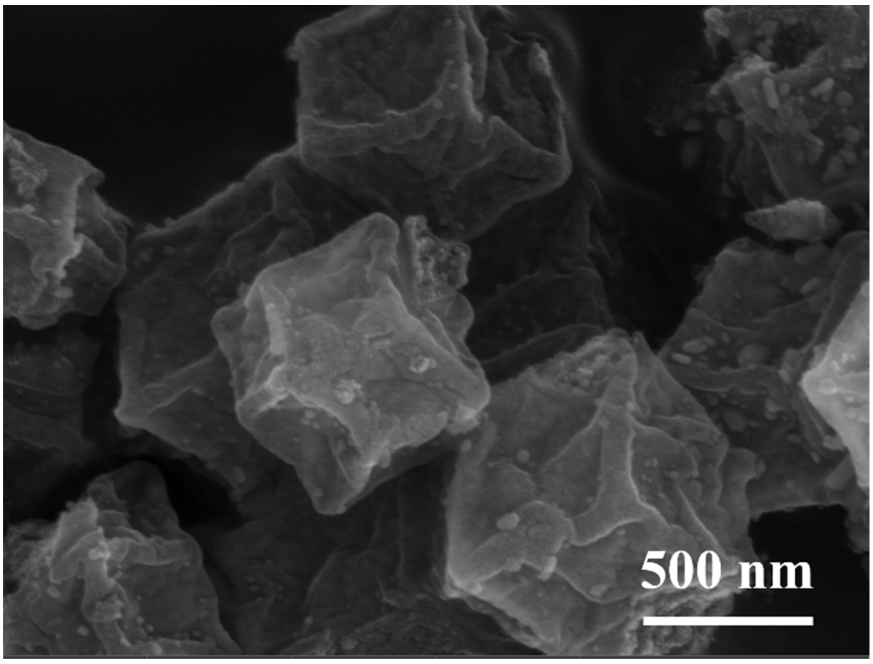

Figure S7:SEM image of the $CoSe_2$-NC NPs.

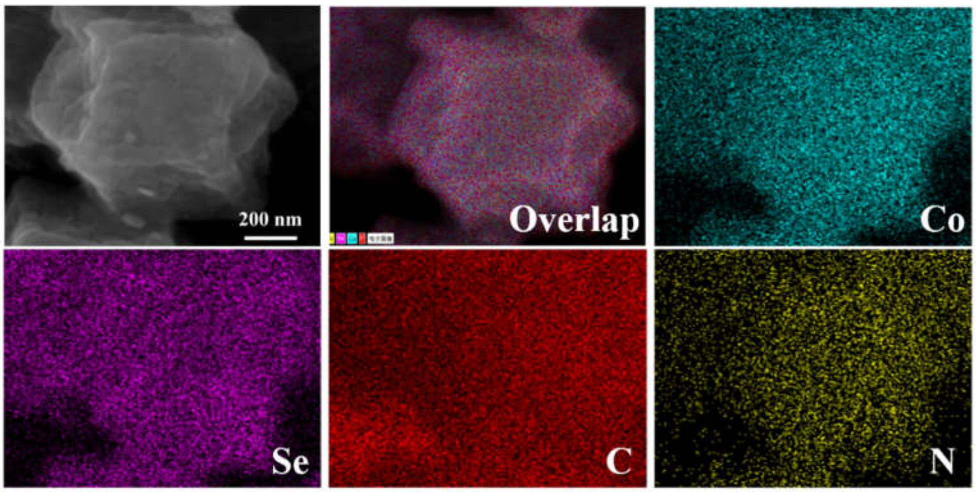

Figure S8: EDS elemental mapping images of $CoSe_2$-NC NPs.

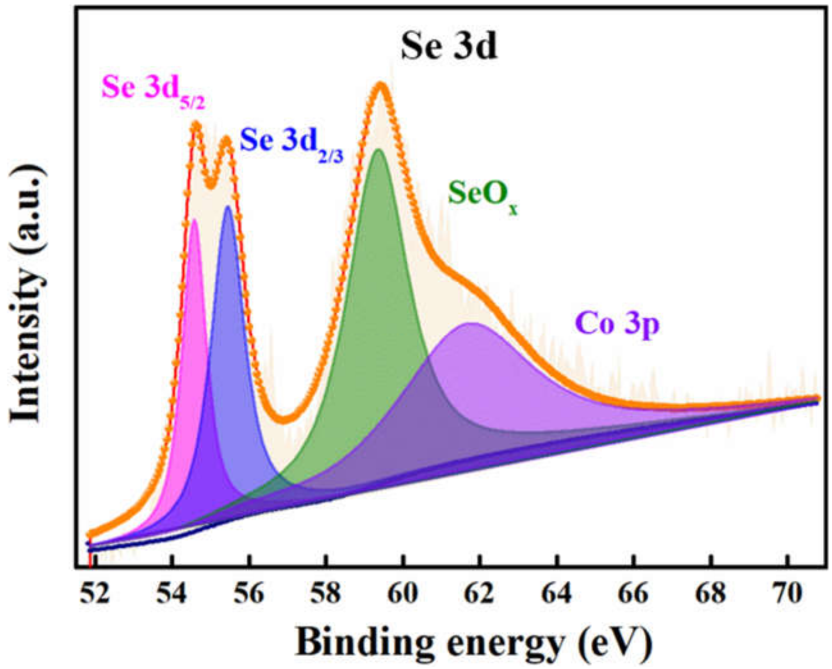

Figure S9**:** High-resolution XPS spectra of Se 3d for the $CoSe_2$-NC NSs.

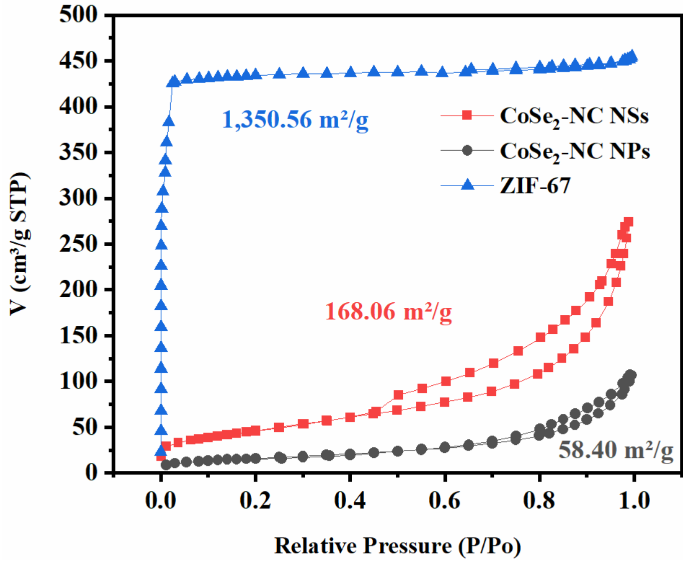

Figure S10: BET surface area of $CoSe_2$-NC NSs, $CoSe_2$-NC NPs and ZIF-67.
